# A pilot study of hot-wire, ultrasonic and wedge-bellows spirometer inter- and intra-variability

**DOI:** 10.1186/s13104-017-2825-0

**Published:** 2017-10-10

**Authors:** Marit E. Aardal, Lene L. Svendsen, Sverre Lehmann, Tomas M. Eagan, Ingvild Haaland

**Affiliations:** 1 Department of Thoracic Medicine, Haukeland University Hospital, 5021 Bergen, Norway; 2 Department of Clinical Science, University of Bergen, Bergen, Norway

**Keywords:** Spirometry, Spirometer comparison, Ultrasonic, Hot-wire, Wedge-bellows

## Abstract

**Objective:**

The aim of this pilot study was to compare spirometric values obtained with different types of spirometers, spirometers of same type, and repeated measurements with the same spirometer in a pulmonary function laboratory setting.

**Results:**

12 healthy volunteers performed spirometry on four hot-wire (SensorMedics), two ultrasonic (Spirare) and one wedge-bellows (Vitalograph S) spirometers, according to ATS/ERS (American Thoracic Society/European Respiratory Society) guidelines. Spirometric values were compared using linear mixed models analysis with a random intercept for subjects and a fixed effect for type of spirometer used. Confidence intervals and *p* values were adjusted for multiple comparisons. Mean ± SD (L) values for hot-wire, ultrasonic and wedge-bellows spirometers for FVC (forced vital capacity) were 4.02 ± 0.66, 3.69 ± 0.61 and 3.93 ± 0.69, and for FEV1 (forced expiratory volume in one second) 3.06 ± 0.44, 2.95 ± 0.44 and 3.10 ± 0.49. Significant differences were found between hot-wire and ultrasonic and between wedge-bellows and ultrasonic spirometers for FVC and FEV1, and between hot-wire and wedge-bellows spirometers for FVC but not for FEV1. There were no significant differences between spirometers of same type, and low mean differences in repeated measurements for all spirometers included. In conclusion, the pilot study shows systematically higher values for FVC and FEV1 for hot-wire and wedge-bellows compared to ultrasonic spirometers.

## Introduction

Spirometry is an important tool to assist diagnosis, detect severity of lung disease, follow disease development, determine effects of and changes in treatment, and to assess preoperative risk [1]. Thus, accurate and reliable results from spirometry testing are necessary for optimal treatment of most patients with lung disease. Multiple factors are known to influence lung function testing, including conditions involving the patient, the instructor and the equipment used [2–7]. With the variety of spirometric equipment used across and within laboratories, differences in measurements between spirometers can be a challenge in patient diagnostics and follow-up, and in research studies. Furthermore, despite meeting the ATS/ERS recommendations when tested with a standard forcing function like a waveform generator, spirometers may exhibit differences in measurements when applied to patients in the clinics. Our aim was therefore to compare the spirometer-subject system performance for different spirometers in a pulmonary function laboratory setting. We compared spirometric values from healthy subjects obtained with different types of spirometers, spirometers of same type, and repeated measurements with the same spirometer.

## Main text

### Methods

We performed a quality assurance study at the Respiratory Physiology Laboratory, Haukeland University Hospital, Bergen, where 12 healthy volunteers performed spirometry on 7 different spirometers within a period of 14 days in 2012. The study was remit assessed by the Regional Ethics Committee (REK Vest; http://helseforskning.etikkom.no, Norwegian Ministry of Education and Research), which classified the study as quality assurance (REK Vest #2016/1552). The spirometers used were four hot-wire (Vmax Encore 22D, Carefusion, Vmax Encore 22D, Vmax Encore 22, Vmax Spectra 229, SensorMedics, referred to as HW1-HW4), two ultrasonic (Spirare SPS320 sensors, Diagnostica AS, referred to as US1 and US2), and one wedge-bellows (Vitalograph S, Vitalograph™ Limited, referred to as WB). All spirometers meet the ATS/ERS recommendations. The hot-wire and wedge-bellows spirometers were calibrated and verified daily with 3-L (hot-wire) or 1-L (wedge-bellows) certified calibration syringes. The ultrasonic spirometers were pre-calibrated from the company, but volume-checked in quality control mode. All spirometers were further volume-checked by testing 3-L calibration syringes in patient mode, where the calibration syringe was completely discharged in one second. Spirometry was conducted by four experienced instructors according to ATS/ERS guidelines [8]. The tests were distributed equally among the four instructors, and both instructors and order of spirometers tested were randomized. Differences in repeated measurements with a spirometer were defined as differences between best and second best measurement in one test. Spirometric values were compared using linear mixed models analysis with a random intercept for subjects and a fixed effect for the type of spirometer used. Repeated covariance structure was set to unstructured to allow for heteroscedasticity. 95% confidence intervals and *p* values were adjusted for multiple comparisons (Bonferroni correction). Bland–Altman plots with 95% limits of agreement were used to demonstrate systematic differences [9]. Statistical analysis was performed using IBM SPSS Statistics, version 24. The level of significance was set at 0.05. Figures were made using Graphpad Prism, version 6.0.

### Results

**Fig. 1:**
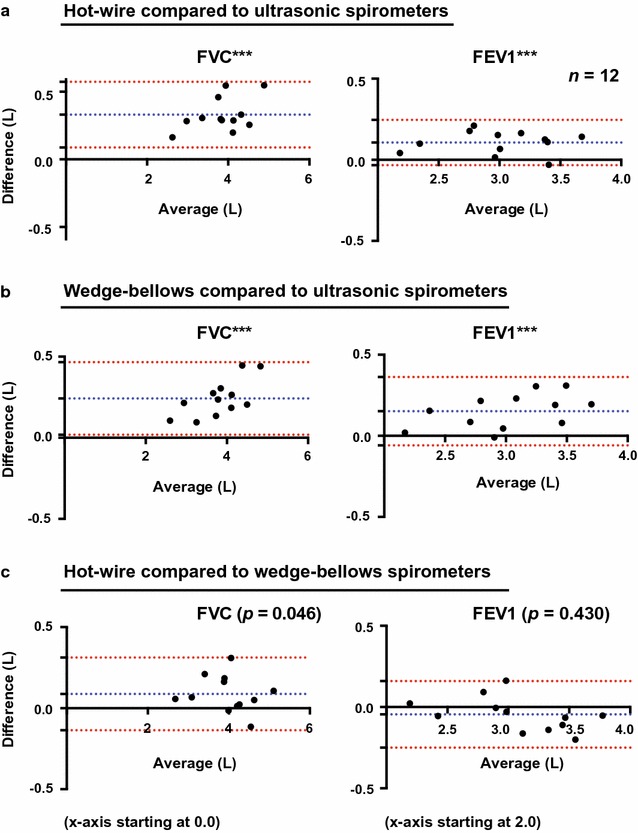
Differences in measurements between hot-wire, ultrasonic and wedge-bellows spirometers for FVC and FEV1. Bland–Altman plots with mean difference ± 1.96SD (95% limits of agreement) for hot-wire compared to ultrasonic spirometers (**a**), for wedge-bellows compared to ultrasonic spirometers (**b**), and for hot-wire compared to wedge-bellows spirometers (**c**). ***p < 0.001, *n.s.* not significant. *p* values are derived from linear mixed models analysis with the spirometer as fixed effect and a random intercept by subject, and are adjusted for multiple comparisons (Bonferroni correction). Significance level is set at 0.05

**Fig. 2:**
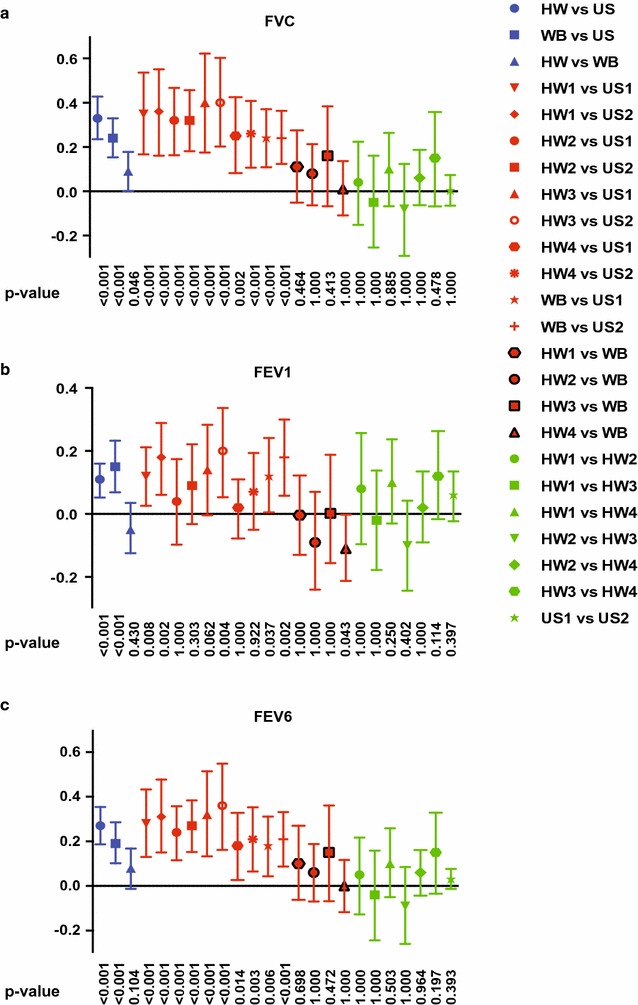
Linear mixed models analysis for comparison of FVC and FEV1 values obtained with seven different spirometers. Mean differences with Bonferroni corrected 95% confidence intervals and *p* values from linear mixed models analysis are shown for comparison of FVC (**a**) and FEV1 (**b**) and FEV6 (**c**) for seven different spirometers; 4 hot-wire (HW1–HW4), two ultrasonic (US1–US2) and one wedge-bellows (WB). Linear mixed models analysis was performed with a random intercept for subjects and a fixed effect for the type of spirometer used. A model with 3 modalities (HW, US, WB) was used for group wise comparisons of the different types of spirometers, and a model with 7 modalities (HW1, HW2, HW3, HW4, US1, US2, WB) was used for comparisons of all spirometers against each other. This set up was used for FVC, FEV1 and FEV6, resulting in six different linear mixed models. Bonferroni corrections were performed for 95% confidence intervals and *p* values for each model. Significance level is set at 0.05

Spirometric values were compared for the different groups of spirometers; hot-wire (HW), ultrasonic (US), and wedge-bellows (WB), and results for FVC and FEV1 are shown by Bland–Altman plots in Fig. 1. Mean ± SD (L) values for HW, US and WB spirometers for FVC were 4.02 ± 0.66, 3.69 ± 0.61 and 3.93 ± 0.69, and for FEV1 3.06 ± 0.44, 2.95 ± 0.44 and 3.10 ± 0.49, respectively. Linear mixed models analysis demonstrated significant differences between HW and US for FVC (*p* < 0.001) and FEV1 (*p* < 0.001), between WB and US for FVC (*p* < 0.001) and FEV1 (*p* < 0.001), and between HW and WB for FVC (*p* = 0.046), but not for FEV1 (*p* = 0.430). Mean differences, mean relative differences and Bland–Altman 95% limits of agreement for FVC and FEV1 are shown in Table 1. No significant differences were found between same type of spirometer (see Table 1). Mean ± SD (L) values for HW, US and WB spirometers for FEV6 were 3.91 ± 0.63, 3.63 ± 0.58 and 3.83 ± 0.65, with significant differences between HW and US (*p* < 0.001) and between WB and US (*p* < 0.01), but not between HW and WB (*p* = 0.104). Mean ± SD (L/min) values for PEF were 478 ± 79, 489 ± 99 and 479 ± 77, respectively (no significant differences). Mean differences with Bonferroni corrected 95% confidence intervals and *p* values from the linear mixed models analyses are shown for FVC, FEV1 and FEV6 for comparisons of different types of spirometers and spirometers of same type (Fig. 2). For differences between best and second best measurement in one test, mean differences were ranging from 0.03 to 0.06 L (0.63–1.51%) for FVC and from 0.03 to 0.05 L (0.84–1.70%) for FEV1 for the different spirometers (Table 1). Results from testing 3-L syringes in patient mode, showed an average FVC of 3.05 L for Spirare sensors, 3.30 L for the Vitalograph, and 3.37 L for the hot-wire spirometers.

**Table 1:** Differences in measurements between different types of spirometers, spirometers of same type, and repeated measurements with a spirometer

Spirometers	FVC			FEV1		
	Mean diff. (L)	Mean rel. diff. (%)	Bland–Altman 95% lim. agr.	Mean diff. (L)	Mean rel. diff. (%)	Bland–Altman 95% lim. agr.
HW–US	0.33***	8.99	(0.09, 0.58)	0.11***	3.59	(− 0.03, 0.24)
WB–US	0.24***	6.56	(0.02, 0.47)	0.15***	5.13	(− 0.06, 0.36)
HW–WB	0.09*	2.28	(− 0.14, 0.31)	− 0.05 n.s.	− 1.46	(− 0.25, 0.16)
HW1–US1	0.35***	9.52	(0.01, 0.69)	0.12**	4.00	(− 0.05, 0.29)
HW1–US2	0.36***	9.64	(− 0.004, 0.72)	0.18**	5.99	(− 0.04, 0.39)
HW2–US1	0.32***	8.53	(0.03, 0.60)	0.04 n.s.	1.29	(− 0.21, 0.29)
HW2–US2	0.32***	8.65	(0.06, 0.57)	0.09 n.s.	3.22	(− 0.14, 0.33)
HW3–US1	0.40***	10.79	(− 0.02, 0.81)	0.14*	4.67	(− 0.13, 0.41)
HW3–US2	0.40***	10.91	(0.03, 0.77)	0.20**	6.67	(− 0.07, 0.46)
HW4–US1	0.25**	6.86	(− 0.06, 0.57)	0.02 n.s.	0.53	(− 0.16, 0.19)
HW4–US2	0.26***	6.98	(− 0.02, 0.54)	0.07 n.s.	2.45	(− 0.15, 0.30)
WB–US1	0.24***	6.50	(− 0.002, 0.48)	0.12*	4.14	(− 0.10, 0.34)
WB–US2	0.24***	6.62	(0.02, 0.47)	0.18**	6.13	(− 0.05, 0.40)
HW1–WB	0.11 n.s.	2.84	(− 0.19, 0.41)	− 0.004 n.s.	− 0.13	(− 0.24, 0.23)
HW2–WB	0.08 n.s	1.91	(− 0.18, 0.33)	− 0.09 n.s.	− 2.74	(− 0.37, 0.20)
HW3–WB	0.16 n.s.	4.02	(− 0.26, 0.58)	0.02 n.s.	0.51	(− 0.31, 0.33)
HW4–WB	0.01 n.s.	0.34	(− 0.21, 0.24)	− 0.11*	− 3.46	(− 0.30, 0.09)
HW1–HW2	0.04 n.s.	0.91	(− 0.31, 0.38)	0.08 n.s.	2.68	(− 0.25, 0.41)
HW1–HW3	− 0.05 n.s.	− 1.14	(− 0.43, 0.34)	− 0.02 n.s.	− 0.64	(− 0.31, 0.27)
HW1–HW4	0.10 n.s.	2.49	(− 0.21, 0.41)	0.10 n.s	3.45	(− 0.14, 0.35)
HW2–HW3	− 0.08 n.s.	− 2.04	(− 0.47, 0.30)	− 0.10 n.s.	− 3.23	(− 0.36, 0.16)
HW2–HW4	0.06 n.s.	1.56	(− 0.17, 0.29)	0.02 n.s.	0.75	(− 0.19, 0.23)
HW3–HW4	0.15 n.s.	3.67	(− 0.25, 0.54)	0.12 n.s	4.12	(− 0.14, 0.38)
US1–US2	0.004 n.s.	0.11	(− 0.1, 0.13)	0.06 n.s.	1.91	(− 0.09, 0.20)

Repeatability	FVC			FEV1		
	Mean diff. (L)	Mean rel. diff. (%)	ATS/ERS approved	Mean diff. (L)	Mean rel. diff. (%)	ATS/ERS approved
HW1	0.05	1.31	Yes	0.05	1.70	Yes
HW2	0.03	0.63	Yes	0.03	1.03	Yes
HW3	0.06	1.51	Yes	0.04	1.30	Yes
HW4	0.04	1.09	Yes	0.03	1.15	Yes
US1	0.04	1.07	Yes	0.03	1.07	Yes
US2	0.05	1.35	Yes	0.05	1.62	Yes
WB	0.03	0.64	Yes	0.03	0.84	Yes

Repeatability: difference between best and second best test, Mean difference: e.g. HW–US, Mean relative difference: e.g. HW–US/US*100

*diff* difference, *HW* hot-wire, *lim. agr.* limits of agreement, *n.s.* not significant, *rel* relative, *US* ultrasonic, *WB* wedge-bellows

* *p* < 0.05, ** *p* < 0.01,*** *p* *<* 0.001 (Bonferroni-corrected from linear mixed models, *n* = 12)

### Discussion

The observed differences in measurements for FVC and FEV1 between the different types of spirometers could be caused by several factors. The SensorMedics instruments are flow-measuring devices with a mass flow sensor based on Kelvin-sensed hot-wire anemometer principles, the Spirare sensors use two-way ultrasound transit time to measure the speed of the airflow, and the Vitalograph measures volume directly by the use of a wedge-bellows [10]. The hot-wire and wedge-bellows spirometers were calibrated and verified daily with different calibration syringes, which may affect the accuracy of the spirometer [11]. The ultrasonic spirometers were pre-calibrated from the company, a procedure previously proved sufficient to retain long-term accuracy in comparable instruments [12].

Spirare sensors apply a fixed BTPS (body temperature and pressure, saturated) correction factor of 1.02 to exhaled air, while the hot-wire instruments apply real-time BTPS correction in which exhalation temperature is measured continuously. The Vitalograph has a fixed BTPS correction factor of 1.09 at 22 °C (used in this study), with additional manual correction for ambient conditions outside of normal range (similar to real-time correction). Results from testing 3-L syringes in patient mode for the different spirometers were within expected values based on the spirometers different BTPS correction systems. When ultrasonic sensors are used for testing patients, exhaled air is measured close to the mouth and there are no physical obstacles in the air channel. Thus, one could argue that variable cooling of air does not affect the results considerably. However, the real-time BTPS correction applied by the hot-wire spirometers also takes into account variations in ambient temperature, pressure and humidity, possibly leading to increased accuracy.

There were also differences in the technique for performing spirometry. For the hot-wire spirometers, 3–4 times of tidal volume measurements were performed before maximal inspiration and forced expiration. For ultrasonic and wedge-bellows spirometers, maximal inspiration was performed before the patient connected to the mouthpiece. There was a tendency to increased difference with increased lung volumes for FVC (see Bland–Altman plots) and FEV6 for hot-wire compared and wedge-bellows compared to ultrasonic, but not for hot-wire compared to wedge-bellows spirometers. Furthermore, there was only a small difference in FVC for hot-wire compared to wedge-bellows spirometers, and no difference in FEV6. These small differences could be caused by dissimilarities in the technique for performing spirometry, while the larger proportional biases for hot-wire and wedge-bellows compared to ultrasonic spirometers are more likely to be caused by differences in measurements principles between the spirometers.

Patient and instructor variability may also interfere with the measurements [3, 4]. Nevertheless, the systematically higher values for FVC and FEV1 for hot-wire and wedge-bellows compared to ultrasonic spirometers suggest that there are actual differences in measurements between the different types of instruments. Similar, but smaller differences have been observed in previous studies of other hot-wire and ultrasonic spirometers [5, 6]. A strength of our pilot study is the inclusion of the Vitalograph, which is considered a gold standard in spirometry testing as it measures volume directly [13]. The Vitalograph has also shown agreement with other types of spirometers, like the pneumotachograph [14].

In conclusion, the pilot study shows systematically higher values for FVC and FEV1 for hot-wire and wedge-bellows compared to ultrasonic spirometers. Technicians and physicians involved in lung function testing and interpretation should be aware of the possible inter-variability between spirometers. The findings should be investigated in larger data sets including patients, instructors and spirometers within and across laboratories. The impact of spirometer inter-variability on conclusions regarding the patient’s diagnosis and treatment should be explored. Furthermore, the results warrant discussion on standardization of BTPS correction in order to improve agreement between spirometers.

## Limitations

The pilot study demonstrates differences in measurements between spirometers for a small data set of 12 healthy individuals. In order to draw conclusions, larger cohorts including different groups of patients with a broad range of spirometric values should be investigated. Spirometry is a physiological test, where the testing procedure and results are influenced by a number of different factors. We have not tested the performance of the spirometers with a waveform generator, as this equipment is not available at our laboratory. However, all spirometers were tested by the manufacturer and shown to meet the ATS/ERS recommendations. Moreover, we could have tested each subject at several different time points for each spirometer to assess the variability within each subject. The instructor conducting the tests adds another well-known source of variability. In larger comparison studies, it is not possible for one instructor to perform all tests. A randomized design for instructors in addition to order of spirometers tested is therefore crucial. Differences in techniques for performing spirometry for the different spirometers may also influence the results, and make it difficult to distinguish between differences in measurements caused by the technique compared the detection itself. Finally, in our mixed models analysis, we have not included adjustments for sex, age and height, as this is a small quality assurance study where these data were not available. These characteristics, together with size of lung volume, are also shown to affect the magnitude of the bias in spirometer comparison studies [5], and should be included in future studies.

## Abbreviations

ATPS: ambient temperature and pressure, saturated
ATS: American Thoracic Society
BTPS: body temperature and pressure, saturated
ERS: European Respiratory Society
FEV1: forced expiratory volume in one second
FEV6: forced expiratory volume in 6 second
FVC: forced vital capacity
HW: hot-wire
PEF: peak expiratory flow
US: ultrasonic
WB: wedge-bellows
